# Michigan State Board of Health

**Published:** 1881-05

**Authors:** 


### ARTICLE VIII.

MICHIGAN STATE BOARD OF HEALTH. Reported for THE CHICAGO MEDICAL JOURNAL AND EXAMINER.

The regular quarterly meeting of this board was held at Lansing, Tuesday, April 12, the following members being present: Rev. D. C. Jacoke, of Pontiac; Henry F. Lyster, M.D., of Detroit; Arthur Hazlewood, M.D., of Grand Rapids; and Henry B. Baker, M.D., Secretary. Dr. Lyster was elected President *pro tem.*

A letter from Prof. Kedzie, President of the board, announced his decision to decline the reappointment as member of the board, for the reason that his duties as professor at the Agricultural college were such as, in the opinion of members of the board of agriculture, would prevent his giving that attention to the work of the board of health which he had heretofore done. His communication outlined the great progress in public health measures in this State since the organization of the State Board of Health eight years ago. He saw with pride that nearly every city, village, and township in the State now has its board of health and health officer. Kerosene explosions, so common eight years ago, have forever been banished. Everywhere in the State there is evidence of an advance in the stamping out of infectious diseases. The ventilation of churches, school houses, and dwellings now receive an attention never known before. The water in our wells, the drainage of farms, and the sewerage of houses have all been brought into prominence by the labors of the board. In this work the board had been greatly assisted by the public press, but the press itself has been stimulated by the work of the board. In short there has been a general advance along the whole line, but we have kept such even step in this advance that we only become aware of our changed position by comparison with the landmarks of eight years ago. Last, but not least, among the agencies set in motion for the public health, he noticed the sanitary conventions for discussion with the people of all matters relating to their physical well-being. He believed they were fraught with inestimable good to the people of our State. The forces which are thus set in motion are not temporary in their influence, but will flow on in a stream of blessings to the end of time. The information gathered by the board needs to be scattered broadcast among the people. New and original investigations into the nature of contagious diseases, and the means for arresting them, need to be undertaken and pushed forward by the board. The information gathered will be of small benefit if imparted to only a few. The State cannot afford to hide this light under a bushel.

In bidding farewell to the State board of health Dr. Kedzie gave the assurance that he did so with the kindliest feelings towards all its members, and with an earnest wish for its highest prosperity and usefulness.

Resolutions were passed expressing extreme regret at the necessity which compelled Dr. Kedzie to decline to serve longer with the board; also, expressing the high appreciation of the board for the eminent labors of Prof. Kedzie in the interests of the public health of the State. The election of his successor as President was postponed until the next meeting of the board.

### THE FILTH OF OUR CITIES.

The Secretary presented a communication from C. H. Voute, giving statistics of the filth removed from privies and cesspools in various places in the State, by means of the odorless excavating apparatus. During the time—about a year—the number of tons removed, is, approximately, as follows: East Saginaw, 850; Bay City, 580; Lansing, 93; Charlotte, 61; Jackson, 151; Ionia, 78; Flint, 118; Battle Creek, 60; Kalamazoo, 258; in the State about 2,300 tons, or 15,000 barrels, and of that amount but 2,000 barrels could be pumped out, the remainder being removed by the "pitting" process, showing the liquid portion had mostly drained off into the soil, which must be much saturated with filth, and as a consequence many wells must be contaminated.

### OIL INSPECTION.

Communications had been received from different parts of the State, stating that it was customary for deputy oil-inspectors to inspect a few barrels of oil from a carload and brand as "approved," and collect pay for inspecting the whole carload. One of the statements was, that the inspector did not test every barrel even when his test showed at least three different grades of oil in the carload. The questions were, whether this was an honest fulfillment of the law, and whether the public safety is thus conserved. The Secretary was directed to take action for ascertaining.

### SICKNESS CAUSED BY PUTRID MEAT.

A letter was presented from John Mulvany, M.D., Surgeon in the British Navy, detailing the effects of food rendered unwholesome through putrefactive taint. All of the crew of a large merchant vessel that put into the Falkland Islands, who ate of pork, opened on a certain day, became ill, and the illness continued until the ship was disabled and medical assistance was sought for in the Falkland Islands. There it was found that not only the pork but the beef was bad, and the meat was condemned by a board of surveying officers. Seven of the affected died, and *post mortem* examination revealed immense effusion into the pericardium, a stench from the brain and congestion at the point of the calamus scriptorious in the fourth ventricle, with congestion of the jejunum and ilium. During life the chief symptoms were paralysis of the hands and feet, and agonizing pain in the toes; uncontrollable sleeplessness, loose bowels, stench from the skin, etc. Symptoms entirely *sui generis.*

The board requested Dr. Mulvany to present a complete account of the sickness.

### DISEASES OF ANIMALS.

A letter was presented from A. J. Murray, U. S. Secretary of the State Cattle Commission, relative to the desirability of collecting statistics of deaths from contagious diseases of animals in all parts of the State. This work might properly have been done by the State Cattle Commission, if it had any funds, but a bill granting them an appropriation of $500, which was passed by the senate was defeated in the house of the present legislature.

Letters were also presented relative to glanders in Clinton and Shiawassee counties.

### SANITARY CONVENTIONS.

Invitations to hold sanitary conventions during the coming winter were accepted from Coldwater and Ann Arbor.

### DETROIT BOARD OF HEALTH.

Dr. Lyster, chairman of the special committee of the board, to devise a plan for a board of health for the city of Detroit, reported that he had in consultation with the city attorney and other citizens drawn up a bill providing a practical and scientific board of health for that city, and the bill was now before the legislature.

### SANITARY SCIENCE EXAMINATIONS.

The annual examination of applicants in sanitary science will be held Tuesday, July 12, 1881; it was voted that the examination should be written, and that each member should submit ten questions not heretofore asked, and on subjects connected with their work as regular committees. Candidates successfully passing the examination will receive certificates that they are qualified to act as health officers in any city, village, or township in the State.

### CONTAGIOUS DISEASES.

It was decided to print revised editions of the documents on the restriction and prevention of each of the three diseases, diphtheria, scarlet fever, and small-pox. Arrangements were also made for the translation of these documents into the Holland and German languages.

### "WINTER CHOLERA."

The Secretary reported the prevalence of a peculiar type of diarrhœa in some portions of the State during the past winter. The fact of its greater prevalence in the southern portions of the State, and that cases have been reported from two State institutions and from towns in the northern part of the State, dependent upon Chicago and Southern Michigan for their food supplies, might indicate a connection between the sickness and the use of oleomargerine, butterine, products of diseased pork, or meat, or other food.

The next regular meeting of the board will be Tuesday, July 13, 1881.

